# Supplementary material for: TRIB3 Mediates Fibroblast Activation and Fibrosis though Interaction with ATF4 in IPF
Source: Int J Mol Sci. 2022 Dec 11;23(24):15705. doi: 10.3390/ijms232415705 (PMC9778945; doi:10.3390/ijms232415705)
Supplement: Supplementary file 1 [file ijms-23-15705-s001.zip › ijms-1970989-supplementary.pdf]

Supplementary materials

Table S1 Clinical information of all patients' samples was used for RT-qPCR and western blot analyses in Fig. 1A. and Fig. 1D.

No.	Gender	Age (year)	Smoking status	Diagnosis
IPF-1	M	69	former	Idiopathic pulmonary fibrosis
IPF-2	M	55	nonsmoker	Idiopathic pulmonary fibrosis
IPF-3	M	53	nonsmoker	Idiopathic pulmonary fibrosis
IPF-4	F	67	former	Idiopathic pulmonary fibrosis
IPF-5	M	62	former	Idiopathic pulmonary fibrosis
IPF-6	F	70	former	Idiopathic pulmonary fibrosis
IPF-7	M	60	nonsmoker	Idiopathic pulmonary fibrosis
IPF-8	F	79	nonsmoker	Idiopathic pulmonary fibrosis
IPF-9	M	75	former	Idiopathic pulmonary fibrosis
IPF-10	F	69	former	Idiopathic pulmonary fibrosis
IPF-11	M	44	former	Idiopathic pulmonary fibrosis
IPF-12	M	71	nonsmoker	Idiopathic pulmonary fibrosis
IPF-13	F	72	former	Idiopathic pulmonary fibrosis
IPF-14	M	60	former	Idiopathic pulmonary fibrosis
IPF-15	M	68	former	Idiopathic pulmonary fibrosis
IPF-16	M	61	nonsmoker	Idiopathic pulmonary fibrosis
IPF-17	M	53	former	Idiopathic pulmonary fibrosis
IPF-18	M	49	former	Idiopathic pulmonary fibrosis
IPF-19	F	67	nonsmoker	Idiopathic pulmonary fibrosis
IPF-20	M	72	former	Idiopathic pulmonary fibrosis
IPF-21	M	76	former	Idiopathic pulmonary fibrosis
IPF-22	F	59	nonsmoker	Idiopathic pulmonary fibrosis
IPF-23	F	51	former	Idiopathic pulmonary fibrosis
CTRL-1	F	70	nonsmoker	NAT lung cancer without fibrosis
CTRL-2	M	60	former	NAT lung cancer without fibrosis
CTRL-3	M	55	nonsmoker	NAT lung cancer without fibrosis
CTRL-4	F	65	former	NAT lung cancer without fibrosis
CTRL-5	F	63	nonsmoker	NAT lung cancer without fibrosis
CTRL-6	M	72	former	NAT lung cancer without fibrosis
CTRL-7	M	68	former	NAT lung cancer without fibrosis
CTRL-8	F	69	nonsmoker	NAT lung cancer without fibrosis
CTRL-9	F	78	former	NAT lung cancer without fibrosis
CTRL-10	F	64	nonsmoker	NAT lung cancer without fibrosis
CTRL-11	M	47	former	NAT lung cancer without fibrosis
CTRL-12	F	70	former	NAT lung cancer without fibrosis

Note: M=Male, F=Female. NAT= adjacent normal tissue

Table S1 Clinical information of all patients' samples was used for RT-qPCR and western blot analyses in Figs. 1A and Fig. 1D.

No.	Gender	Age (year)	Smoking status	Diagnosis
CTRL-13	M	70	nonsmoker	NAT lung cancer without fibrosis
CTRL-14	M	59	former	NAT lung cancer without fibrosis
CTRL-15	F	56	former	NAT lung cancer without fibrosis

Note: M=Male, F=Female. NAT= adjacent normal tissue
